# The Benefits of Forgiveness at Work: A Longitudinal Investigation of the Time-Lagged Relations Between Forgiveness and Work Outcomes

**DOI:** 10.3389/fpsyg.2021.710984

**Published:** 2021-07-27

**Authors:** Wenrui Cao, Reine C. van der Wal, Toon W. Taris

**Affiliations:** Department of Social, Health and Organizational Psychology, Utrecht University, Utrecht, Netherlands

**Keywords:** forgiveness, work outcomes, cross-lagged panel model, interpersonal relationships, well-being

## Abstract

Forgiveness has received increasing attention in the work context. Although recent cross-sectional studies have found a positive link between forgiveness and work outcomes, further research examining the temporal dynamics between these variables is needed to establish causality. This preregistered panel study investigated the time-lagged relations between forgiveness and work outcomes, and specifically addressed the question whether forgiving a coworker benefits work outcomes. Longitudinal survey data were collected at four time points among 139 Chinese employees working at least 20 hours per week. Results from cross-lagged panel models revealed that forgiving an offending coworker with whom one has a relatively good work relationship predicted better work outcomes (i.e., higher job satisfaction, higher work engagement, and lower burnout) over time, while controlling for perceived severity of the offense. Evidence for the reverse effect (with work outcomes predicting forgiveness) was not found. Our findings thus suggest that forgiveness facilitates well-being-related work outcomes. Implications for a better understanding of forgiveness in work relationships are discussed.

## Introduction

Interpersonal mistreatment is a common issue experienced by employees around the globe (Schilpzand et al., 2016). To illustrate, in South Korea, 70% of the employees indicated to have been bullied by their work superiors and colleagues in their working life, with around 12% enduring bullying on a daily basis (National Human Rights Commission, 2019). These offenses can be harmful to both employees and organizations, and may result in reduced performance, increased deviant behaviors, and impaired mental and physical health (e.g., Bowling and Beehr, 2006). How to deal with these offenses properly and hence mitigate their harmful consequences for employees, organizations and even society is a major concern for all parties involved.

Social scientists have recognized the potential beneficial role of forgiveness in dealing with the offenses that inevitably take place in interpersonal relationships (c.f., Fincham, 2000). Defined as a prosocial change toward the offender despite the offender’s hurtful actions (e.g., McCullough et al., 1998; McCullough, 2001), forgiveness has been conceptualized both as a general tendency (i.e., trait forgiveness) and following a specific transgression (i.e., state forgiveness). In this study we focused on forgiveness as a response to a specific transgression (i.e., state forgiveness; hereafter referred to as *forgiveness*). It has been shown that forgiveness is associated with better psychological and physical well-being (Karremans et al., 2003), and increased relationship satisfaction (e.g., McCullough et al., 1998; Karremans and Van Lange, 2008). Importantly, recent research suggests that forgiveness might also be associated with better work outcomes, such as higher job satisfaction (e.g., Cox, 2011; Stackhouse, 2019). Yet, existing evidence on the association between forgiveness and outcomes in the workplace is exclusively based on cross-sectional studies, leaving the question unaddressed whether forgiveness facilitates better work outcomes, and/or vice versa. Given the many ways in which forgiveness may potentially benefit both individual and organizational outcomes (e.g., Lundahl et al., 2008; Wade et al., 2014), it is important to address the causality question. Therefore, using a longitudinal design, this research examined the causal relationship between interpersonal forgiveness among employees and their work outcomes.

### Forgiveness and Work Outcomes

Why would forgiveness among employees be associated with better individual work outcomes? To address this question, it is important to consider the relationship context in which forgiveness (or the lack thereof) is taking place. Despite the findings that higher-quality work relationships (i.e., relationships defined in terms of mutual respect, trust, and obligation between employees; Graen and Uhl-Bien, 1995) promote forgiveness tendencies (Cao et al., in press), employees may not always feel capable of responding with forgiveness when offended, even when the offender is someone they respect and trust. In some cases employees may avoid the coworker (e.g., by reducing their collaboration; Hershcovis et al., 2018), but there may also be moments, particularly in stable work relationships, when an employee wants to maintain the relationship despite of what happened (e.g., Radulovic et al., 2019). At some point, employees may thus find themselves in a situation in which they have a good work relationship with a coworker, while simultaneously they are having a hard time forgiving the offending coworker.

The lack of forgiveness toward a “good colleague” (having a relatively good relationship quality with) may undermine work outcomes in at least two ways. As suggested in previous research (e.g., Rothmann, 2008), work outcomes can be seen as a broad category of phenomena that includes job satisfaction, work engagement, and burnout. First, a lack of forgiveness may be associated with retaliatory and aggressive responses that may deteriorate the work relationship (Bradfield and Aquino, 1999). Research indicates that employees using hostile and aggressive conflict strategies in responses to workplace offenses have less stable work relationships and are less accepted by their coworkers (e.g., Kim et al., 2008). Unstable work relationships negatively affect employees’ work outcomes, such as leading to reduced work engagement (Weigl et al., 2010; Liao et al., 2013). Hence, given the otherwise beneficial outcomes of good work relationships, employees’ inability to forgive may undermine their work outcomes because this inability deteriorates crucial work relationships.

Second, and relatedly, employee’s lack of forgiveness toward a coworker may increase stress and tension. Indeed, previous research has demonstrated that the combination of a lack of forgiveness on the one hand, and the motivation to maintain a good relationships (in this case, a romantic relationship) on the other hand, can contribute to a state of psychological tension (Karremans et al., 2003; Kluwer and Karremans, 2009). Increased tension strongly affects individual work outcomes (Cordes and Dougherty, 1993), such as reduced job satisfaction and increased burnout (Volmer and Wolff, 2018), and absenteeism (Hees et al., 2013). Psychological tension created by these competing motives may thus negatively affect work outcomes.

Previous research indeed reveals that a lack of forgiveness is associated with lower job satisfaction (Law, 2013; Radulovic et al., 2019), lower work engagement (Little et al., 2007), less commitment to the organization (Basford et al., 2014), more burnout (Booth et al., 2018; Hershcovis et al., 2018) and higher levels of job stress (Cox, 2011). However, almost all evidence regarding the association between forgiveness and work outcomes relies on cross-sectional designs. As far as we know, only one study by Stackhouse (2019) demonstrated that more forgiveness predicted higher job satisfaction and lower intentions to leave across a two-week interval. Although interesting, this research did not take into account the causal effect of (state) forgiveness and various work outcomes.

Furthermore, given the lack of evidence on the causal role of forgiveness in an organizational context, we draw from studies in close relationships (i.e., friendships, romantic relationships, and family relationships; e.g., McCullough et al., 1997; Fincham et al., 2004) in understanding the causal role of forgiveness on work outcomes. Specifically, longitudinal and experimental studies in close relationships found forgiveness to be causally related to both intra-personal outcomes, such as enhanced psychological and physical well-being (McCullough et al., 2001; Bono et al., 2008; Seawell et al., 2014), as well as interpersonal outcomes, such as enhanced relationship satisfaction, commitment, and stability (Paleari et al., 2005; Tsang et al., 2006; Braithwaite et al., 2011). For example, Bono et al. (2008) found that forgiveness was causally related to more positive mood, fewer negative mood and fewer physical symptoms. Similarly, not being able to forgive an offending other was prospectively associated with declines in physical health three years later (Seawell et al., 2014). These findings correspond with meta-analytic evidence on forgiveness interventions, revealing that participants who had received a forgiveness intervention displayed fewer depressive and anxiety symptoms as well as greater levels of hope than no-treatment control conditions (Wade et al., 2014). Moreover, Tsang et al. (2006) found that forgiveness predicted more closeness and commitment toward romantic partners and friends two weeks later. Finally, forgiving a romantic partner was associated with increased relationship satisfaction over a two-month interval (Braithwaite et al., 2011). Taken together, we hypothesize that:

*H1a*: Forgiveness in high-quality work relationships is associated with better work outcomes at a later point in time.

It is also possible that work outcomes causally predict higher levels of forgiveness. Following the reasoning above, better work outcomes (such as job satisfaction and work engagement) may reversely increase an employees’ individual well-being as well as their work relationships (e.g., Volmer et al., 2011). However, as compared to the reversed causal pattern (with forgiveness causally predicting work outcomes), only a handful studies found that individual or relational well-being predicted more forgiveness across time. Specifically, Bono et al. (2008) found that increases in psychological well-being were causally related to increases in forgiveness two weeks later. Moreover, higher relationship quality was associated with more forgiveness several years later (Paleari et al., 2005; Fincham and Beach, 2007). In sum, although the evidence is limited, in this study we also investigated the possibility of a reversed causal association that work outcomes predict more forgiveness. We therefore include the hypothesis that:

*H1b*: Higher work outcomes are associated with more forgiveness in high-quality work relationships at a later time point.

### The Present Research

The present research used a longitudinal design to address the question whether forgiveness in high quality work relationships predicts better work outcomes (i.e., job satisfaction, work engagement, and burnout). Measuring these variables over multiple time points enabled us to investigate the direction of potential causal effects between forgiveness and work outcomes. In this study, we considered the following restrictions and control variables. First of all, given the importance of work relationship quality in understanding the association between forgiveness and general work outcomes, we exclusively focused on forgiveness in relatively high-quality work relationships. Moreover, since perceived severity of the offense is generally negatively associated with forgiveness (Fehr et al., 2010), we controlled for perceived severity of the offense in our analyses. Finally, in line with previous research revealing that the relative status between victim and offender influences forgiveness (e.g., Aquino et al., 2001; Bies et al., 2016; Zheng and van Dijke, 2020), we also took into account the status difference between victim and offender (Aquino et al., 2006). All data scripts and materials can be viewed at the Open Science Framework by following this link: bit.ly/3ps7KdJ.

## Materials and Methods

### Participants

Participants were recruited in China^1^ through Credamo, a professional Chinese platform for online data collection. Individuals aged at least 18 years old, working at least 20 h per week, and working in a team with at least three other members were invited to participate in the longitudinal study. The study consisted of four time points (T1-T4), with a one-week interval between each time point (for a similar procedure, see McCullough et al., 2003). At Time 1, 527 eligible participants took part in the study, three participants indicated that we should not use their data, and another 27 participants failed to follow our instruction to recall a hurtful incident by a coworker. Data were available for 497 employees at Time 1, 139 employees at Time 2, 138 employees at Time 3 and 130 employees at Time 4. As a result, 139 participants with full data on two or more time points were entered in the analyses. To investigate the potential impact of attrition, we tested mean-level differences on our key variables (forgiveness, job satisfaction, work engagement, and burnout) at Time 1 between participants who completed all four time points and participants who dropped out of the study after Time 1 (*N* = 358). Independent *t*-tests revealed no significant differences for any of the variables (see Appendix A), suggesting that our final sample was generally representative for the larger sample that started the study.

Participants (56.1% female) were 19 to 53 years old (*M* = 30.88, *SD* = 6.19), and mostly held a university degree (77.7%). On average, they worked 47.76 h per week (*SD* = 8.26) in a broad variety of industries. The average number of working years in their current organizations was 5.25 years (*SD* = 5.03) and average team tenure was 2.23 years (*SD* = 1.93). When asked to indicate at which level they were working in the organization (1 = the lowest level, 10 = the highest level), 69.8% participants indicated to be working in a higher position (higher than mean level of 5). Participants received 28 yuan (about €3.50) for their participation in all four time points.

### Procedure

The study was approved by the Ethics Committee of our institution and preregistered at aspredicted.org (bit.ly/37o9RZW). This study was part of a larger study in which we investigated the developmental trajectory of forgiveness in the workplace. For this specific study we focused on the causal relationship between forgiveness and work outcomes. Given that only 139 of the intended 360 participants completed the entire study, we slightly deviated from our pre-registered plan and did not test for mediations by general health and team-member exchange. Items within scales were presented randomly.

At Time 1 (T1), after providing informed consent and demographic information, participants were asked to recall and describe a hurtful incident by one of their coworkers including the following restrictions: (1) The hurtful incident took place in the workplace; (2) The hurtful incident took place in the past seven days; (3) The offender was someone the participant had a good work relationship with; (4) The participant felt or still feels hurt by the hurtful incident; and (5) It was the other to blame (at least in the perspective of the participant). An example description of a hurtful incident was “It happened the day before yesterday, our company checked the quality of work, a colleague who has a good work relationship with me picked various problems on me. I felt like he was taking shots at me, which made me very faceless and uncomfortable.” Next, they received some questions about the incident. As preregistered, we removed participants who did not follow the instructions (i.e., did not recall a hurtful incident by a close other that took place in the past seven days, *N* = 27). Participants also received questions about their level of forgiveness, and their work outcomes. At the following time points (T2–T4), participants were presented with the same incident (by uploading a screen shot of the description of the incident they recalled at Time 1, and asked to read it carefully again. Next, they completed the same questions regarding the incident, forgiveness, and work outcomes as they did at Time 1. After completing the survey, participants were thanked and debriefed.

### Measures

All items were presented in Chinese. Unless reported otherwise, participants responded to items on 7-point Likert scales ranging from 1 (strongly disagree) to 7 (strongly agree). The internal consistency (Cronbach’s alpha) of the multi-item measures varied from 0.82 to 0.96 (cf. Table 2), and *M*_*alpha*_ was 0.89.

**TABLE 1:** Configural and metric invariance variables.

	**Model**	**χ^2^**	***df***	**RMSEA**	**CFI**	**TLI**	**SRMR**
Forgiveness	Configural model	1549.65	972	0.07	0.93	0.92	0.06
	First-order metric model	1573.14	999	0.06	0.93	0.92	0.06
	Second-order metric model	1576.89	1005	0.06	0.93	0.92	0.06
Work Outcomes	Configural model	1262.16	801	0.06	0.92	0.91	0.06
	First-order metric model	1291.24	825	0.06	0.92	0.91	0.07
	Second-order metric model	1312.34	831	0.07	0.92	0.91	0.07

**TABLE 2:** Descriptive statistics and correlations for main study variables.

	**1**	**2**	**3**	**4**	**5**	**6**	**7**	**8**	**9**	**10**	**11**	**12**	**13**	**14**	**15**	**16**	**17**	**18**	**19**	**20**
1. RQ																				
2. Severity	–0.03	(0.86)																		
3. Time	–0.03	0.10																		
4. Offender	0.21*	0.01	−0.19*																	
5. Forg-T1	0.42**	−0.47**	0.04	0.10	(0.95)															
6. Forg-T2	0.41**	−0.42**	0.01	0.11	0.87**	(0.96)														
7. Forg-T3	0.37**	−0.41**	–0.05	0.08	0.79**	0.91**	(0.96)													
8. Forg-T4	0.37**	−0.43**	–0.01	0.07	0.81**	0.90**	0.95**	(0.96)												
9. JS-T1	0.34**	0.02	0.05	0.12	0.30**	0.33**	0.21*	0.24**	(0.84)											
10. JS-T2	0.30**	–0.01	0.06	0.12	0.34**	0.38**	0.30**	0.28**	0.68**	(0.77)										
11. JS-T3	0.38**	0.00	0.16	0.17*	0.32**	0.40**	0.32**	0.36**	0.72**	0.75**	(0.82)									
12. JS-T4	0.41**	–0.06	0.06	0.19*	0.41**	0.43**	0.39**	0.41**	0.68**	0.73**	0.84**	(0.89)								
13. WE-T1	0.36**	0.03	0.06	0.17*	0.34**	0.36**	0.26**	0.24**	0.74**	0.65**	0.68**	0.68**	(0.84)							
14. WE-T2	0.37**	0.05	0.15	0.17*	0.33**	0.38**	0.28**	0.27**	0.68**	0.71**	0.73**	0.69**	0.76**	(0.87)						
15. WE-T3	0.41**	–0.06	0.14	0.22**	0.35**	0.41**	0.31**	0.36**	0.67**	0.61**	0.76**	0.73**	0.75**	0.80**	(0.88)					
16. WE-T4	0.48**	–0.07	0.14	0.17	0.39**	0.42**	0.38**	0.39**	0.67**	0.73**	0.87**	0.85**	0.72**	0.77**	0.85**	(0.88)				
17. BO-T1	−0.35**	0.23**	–0.09	–0.11	−0.43**	−0.45**	−0.40**	−0.34**	−0.52**	−0.52**	−0.54**	−0.57**	−0.55**	−0.55**	−0.53**	−0.58**	(0.90)			
18. BO-T2	−0.25**	0.12	–0.16	–0.03	−0.32**	−0.37**	−0.35**	−0.27**	−0.46**	−0.59**	−0.50**	−0.49**	−0.52**	−0.61**	−0.50**	−0.53**	0.77**	(0.92)		
19. BO-T3	−0.33**	0.12	–0.10	–0.03	−0.32**	−0.39**	−0.40**	−0.36**	−0.46**	−0.56**	−0.61**	−0.60**	−0.49**	−0.51**	−0.57**	−0.64**	0.76**	0.80**	(0.92)	
20. BO-T4	−0.36**	0.14	–0.15	–0.13	−0.38**	−0.42**	−0.41**	−0.39**	−0.39**	−0.50**	−0.57**	−0.59**	−0.47**	−0.53**	−0.56**	−0.61**	0.76**	0.79**	0.84**	(0.91)
*M*	5.40	5.06	4.82	1.83	4.59	4.51	4.62	4.75	5.56	5.59	5.60	5.66	5.22	5.18	5.29	5.42	2.91	2.85	2.65	2.58
*SD*	0.93	1.27	1.81	0.59	1.23	1.25	1.28	1.37	0.98	0.89	0.91	1.01	1.04	1.08	1.03	0.99	1.30	1.28	1.23	1.19

N = 130–139; alphas are reported on the diagonal. *p < 0.05, **p < 0.01 (two-tailed); RQ = Relationship Quality; Forg = Forgiveness; JS = Job Satisfaction; WE = Work Engagement; BO = Burnout; T1–T4 refer to Time 1–Time 4, respectively.

#### Questions About the Hurtful Incident

After recalling the incident, participants received questions related to the incident. They were asked: (1) how long ago the hurtful incident took place (in days); (2) how they rated the quality of their work relationship with the offender *before* the hurtful incident took place (1 = very low, 7 = very high); (3) how severe they thought the incident was (three items, e.g., “The incident was severe”; van der Wal et al., 2014); and (4) whether it was their supervisor (*N* = 38), peer coworker (*N* = 87) or subordinate (*N* = 14) who had offended them.

#### Forgiveness

Forgiveness was assessed using the Transgression-Related Interpersonal Motivations Inventory (TRIM) developed by McCullough et al. (1998), which consists of 3 dimensions: benevolence toward the offender (4 items; e.g., “Despite the incident, I want to have a positive relationship”), revenge (4 items; e.g., “When I think about the incident, I wish that something bad would happen to him/her”) and avoidance (4 items; e.g., “When I think about the incident, I would rather avoid him/her”). We reverse-scored the revenge and avoidance subscales, so that a higher score indicated more forgiveness.

#### Work Outcomes

Three work outcomes (job satisfaction, work engagement, and burnout) were assessed. *Job satisfaction* was measured using a subscale of the Michigan Organizational Assessment Questionnaire (Cammann et al., 1979). Participants were asked to indicate their agreement with three items, including: “Usually, I really enjoy my work.” *Work engagement* was measured with the short version of the Utrecht Work Engagement Scale (UWES-3; Schaufeli et al., 2019) that taps the three core dimensions of work engagement (vigor, dedication, and absorption) with one item for each dimension, e.g., “This week, I felt like going to work when I got up in the morning.” *Burnout* was captured using the 5-item emotional exhaustion subscale of the Maslach Burnout Inventory-General Survey (Maslach et al., 1986). An example is “I feel used up at the end of a work day.”

### Statistical Analysis

Correlational analyses were conducted to obtain basic insight into the data. Structural equation modeling (SEM) was used in Mplus v8.3 (Muthén and Muthén, 1998–2017). All models were evaluated using the chi-square test, the Root Mean Square Error of Approximation (RMSEA) (Browne and Cudeck, 1992), the Tucker-Lewis Index (TLI) (Tucker and Lewis, 1973), the Comparative Fit Index (CFI) (Bentler, 1980), and the Standardized Root Mean Square Residual (SMRM). As there is no consensus on cut-off values for adequate fit (e.g., Lance et al., 2006), conservative guidelines were followed, with fit considered to be acceptable if RMSEA is lower than 0.08, TLI and CFI are 0.90 or higher, and SMRM is 0.08 or lower (Bentler, 1980; Hu and Bentler, 1999).

#### Construct Validity

Consistent with previous research (e.g., Forster et al., 2020), forgiveness was taken as a second-order factor. Confirmatory factor analysis (CFA) of the TRIM scale confirmed the existence of three first-order factors: benevolence, revenge, and avoidance. As suggested by previous research (e.g., Rothmann, 2008) and following our pre-registration, we then checked whether the three work outcomes could be combined into a second-order factor to reflect general work outcomes. We assessed the fit of our data to a measurement model of three work outcome indicators (i.e., job satisfaction, work engagement and burnout). The second-order factor model of work outcomes provided good fit (for Time 1, χ^2^ = 67.73, *df* = 41; CFI = 0.98; TLI = 0.97; RMSEA = 0.07; SMRM = 0.04; for Time 2, χ^2^ = 63.98, *df* = 41; CFI = 0.98; TLI = 0.97; RMSEA = 0.06; SMRM = 0.05; for Time 3, χ^2^ = 85.56, *df* = 41; CFI = 0.96; TLI = 0.95; RMSEA = 0.09; SMRM = 0.05; for Time 4, χ^2^ = 73.61, *df* = 41; CFI = 0.97; TLI = 0.96; RMSEA = 0.08; SMRM = 0.05). We therefore proceeded our analyses using the second-order factor to reflect general work outcomes.

We then conducted four separate CFAs to ensure each survey item was loading appropriately on its respective factor (i.e., second-order factor of forgiveness and second-order factor of work outcomes). The results of these analyses revealed that the hypothesized two-factor second-order factor model provided adequate fit to the data at each time point (for Time 1, χ^2^ = 425.30, *df* = 223; CFI = 0.92; TLI = 0.91; RMSEA = 0.08; SMRM = 0.08; for Time 2, χ^2^ = 431.45, *df* = 223; CFI = 0.93; TLI = 0.92; RMSEA = 0.08; SMRM = 0.05; for Time 3, χ^2^ = 426.31, *df* = 223; CFI = 0.93; TLI = 0.92; RMSEA = 0.08; SMRM = 0.07; for Time 4, χ^2^ = 354.91, *df* = 223; CFI = 0.96; TLI = 0.95; RMSEA = 0.07; SMRM = 0.06). Moreover, the hypothesized two-factor model fitted the data significantly better than a more parsimonious one-factor model in which all the items loaded on a single factor (Δχ^2^_[__7__]_ ranged from 989.53 to 1205.03, all *p*s < 0.01). Overall, these results supported the distinctiveness of our constructs within each time point.

#### Measurement Invariance

We then conducted a series of longitudinal CFAs to check the measurement invariance of our constructs across time (Taris et al., 1998). We started with a configural model, in which we applied the same factor structure across time. A well-fitting configural model would demonstrate that the constructs that are assessed across each measurement time point all tap into the same construct. As shown in Table 1, the configural models provided adequate fit to the data, supporting the assumption that the factor structures of the research variables were consistent across time (Vandenberg and Lance, 2000; Liang et al., 2018).

Next, we tested the metric invariance of the first-order factors (first-order metric model; Rudnev et al., 2018), in which the loadings on the same first-order factors were constrained to be equal across time, and the loadings on the second-order factors were freely estimated. As shown in Table 1, the first-order metric invariance was supported by the data, implying that covariances between the first-order factors were comparable. Therefore, the loadings of the second-order factors can be meaningfully compared across time.

We proceeded by estimating a second-order metric model, in which the loadings were constrained to be equal on the same first-order factors as well as second-order factors across time. As suggested in previous studies (e.g., Brown, 2006), in all measurement models, error variances of the same indicators used across time points were allowed to be correlated to account for their non-independence. The results of the second-order metric model are reported in Table 1, which provided evidence for second-order metric invariance of our constructs over time. However, since the standard errors of the model parameter estimates may not be completely trustworthy due to our relatively small sample size and also because the subject-to-parameter ratio becomes worse when adding longitudinal effects, we decided to proceed our data analysis by treating the constructs as observable variables. That is, we used mean scores for corresponding constructs (Halbesleben, 2010; Liu et al., 2020).

#### Analysis Strategy

**FIGURE 1:**
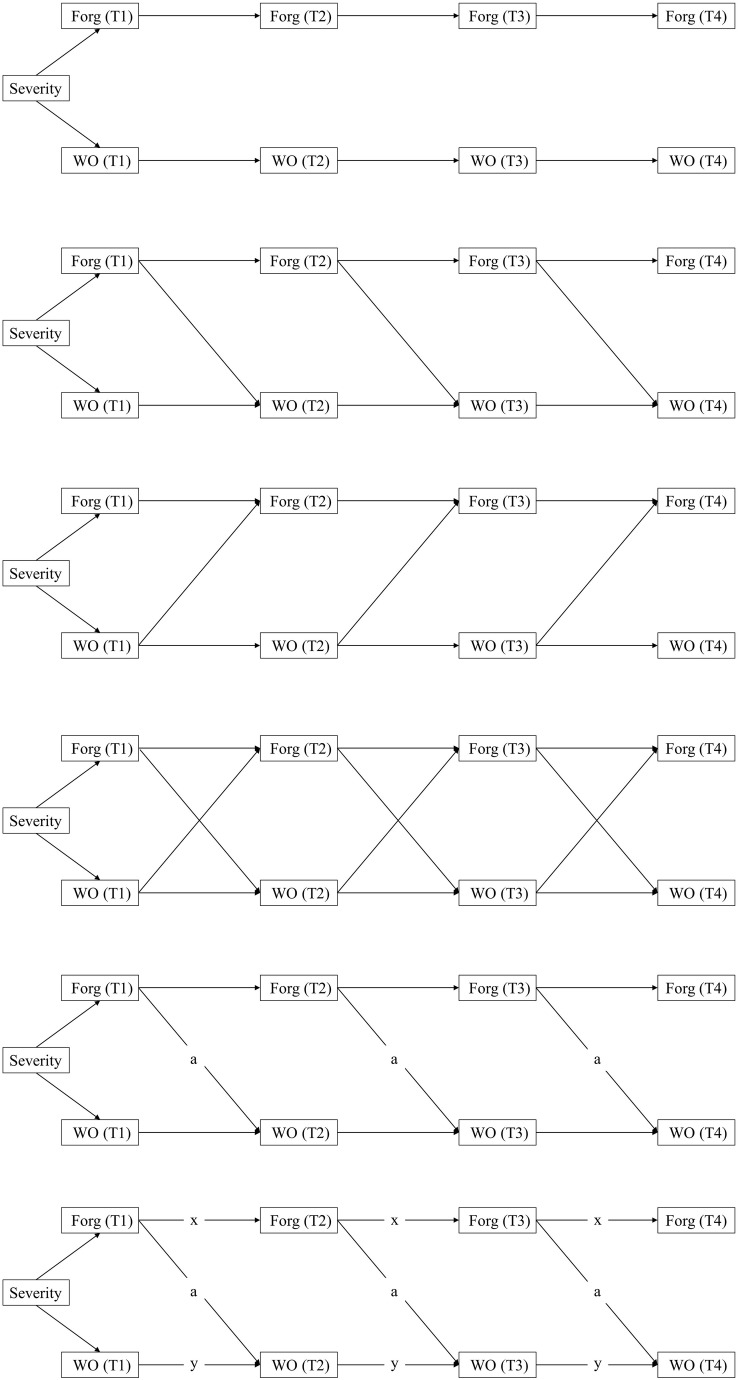
Schematic representation of competing models. Forg = Forgiveness; WO = Work outcomes; T1–T4 refer to Time 1-Time 4, respectively. Paths labeled “x” and “y” estimate stability coefficients. Paths labeled “a” and “b” estimate cross-lagged coefficients.

After confirming the adequacy of construct validity and measurement invariance, we used a cross-lagged panel model (CLPM) to test the dynamic relations among variables with MPlus 8.3 (Muthén and Muthén, 1998–2017). In this model, there are two primary relations of interest (Mund and Nestler, 2019): (a) the auto-regressive relations among the same constructs across time, and (b) the cross-lagged relations among different constructs across time. We fitted four competing path models to our data (see Figure 1): a stability model (M_1_), a forgiveness-to-work outcomes model (M_2_), a work outcomes-to-forgiveness model (M_3_), and a reciprocal model (M_4_). The stability model (M_1_) expresses the stability within each variable over time, and estimates the auto-regressive paths of forgiveness and work outcomes separately, that is, forgiveness at Time *i* was set to predict forgiveness at Time *i* + 1 (*i* = 1, 2, 3), and work outcomes at Time *i* was set to predict work outcomes at Time *i* + 1 (*i* = 1, 2, 3). The forgiveness-to-work outcomes model (M_2_) estimates the lagged-impact of forgiveness on work outcomes after controlling for the stability of forgiveness and work outcomes separately over time. Specifically, based on the stability model (M_1_), we specified the cross-lagged paths from forgiveness (as the explanatory variable) at Time *i* to work outcomes (as the dependent variables) at Time *i* + 1 (*i* = 1, 2, 3). The work outcomes-to-forgiveness model (M_3_) was also based on the stability model M_1_, but included reverse cross-lagged paths compared to M_2_. In other words, we specified the cross-lagged paths from work outcomes (as independent variable) at Time *i* to forgiveness (as dependent variable) at Time *i* + 1 (*i* = 1, 2, 3). Finally, the reciprocal model included cross-lagged paths between Time i forgiveness and Time *i* + 1 (*i* = 1, 2, 3) work outcomes as well as the cross-lagged paths between Time *i* work outcomes and Time *i* + 1 (*i* = 1, 2, 3) forgiveness. We tested whether models with cross-lagged effects (M_2_-M_4_) fitted the data significantly better than the stability model (M_1_). Furthermore, to determine whether these relationships were consistent across time, we computed additional chi-square difference tests that compared unconstrained models to the models that constrained cross-lagged effects and/or auto-regressive effects being the same within the same relationships. Following our pre-registration, we controlled for perceived severity of the hurtful incident when estimating these models.

## Results

### Preliminary Analyses

Means, standard deviations, and intercorrelations for all variables are displayed in Table 2. The correlation coefficients among the same variable measured at different time points (i.e., test–retest reliability) were substantial and significant for forgiveness (*r*s ranging from 0.79 to 0.95, all *p*s < 0.01); job satisfaction (*r*s ranging from 0.68 to 0.84, *p*s < 0.01); work engagement (*r*s ranging from 0.72 to 0.85, *p*s < 0.01) and burnout (*r*s ranging from 0.76 to 0.84, *p*s < 0.01). In line with previous work (McCullough et al., 1998; Burnette et al., 2012), work relationship quality was significantly positively (*r*s ranging from 0.37 to 0.42, *ps* < 0.01), and perceived severity of the incident was significantly negatively (*r*s ranging from −0.47 to −0.41, *p*s < 0.01) associated with forgiveness. Both time since the incident took place (i.e., time) and whether the offender was one’s supervisor, peer coworker or subordinate (i.e., offender) were unrelated to forgiveness. We therefore did not consider offender status as a control variable in subsequent analyses. Most importantly, consistent with previous cross-sectional work (Cao et al., in press), across all four time points, forgiveness was associated with higher levels of job satisfaction (*r*s ranging from 0.21 to 0.43, *p*s < 0.01) and work engagement (*r*s ranging from 0.26 to 0.42, *p*s < 0.01) and lower levels of burnout (*r*s ranging from −0.45 to −0.32, *p*s < 0.01).

### Main Analyses

Table 3 presents the fit indices for the competing models. Except for a relatively high RMSEA, the other fit indices of all models were acceptable (CFI ≥ 0.90; TLI ≥ 0.90; SMRM ≤ 0.80; see Table 3). The chi-squared difference tests in Table 4 showed that except for M_3_, both M_2_ and M_4_ improved significantly on the stability model M_1_. Moreover, the results revealed no significant difference between M_2_ and M_4_ [Δχ^2^(3) = 6.44, *p* = 0.09], thus the more parsimonious model (M_2_) was retained for further analysis. M_2__*a*_ constrained the cross-lagged effects to be equal over time based on M_2_, and M_2__*b*_ constrained auto-regressive effects being equal over time based on M_2__*a*_ (see Figure 1). The difference between the unconstrained model M_2_ and its constrained counterpart M_2__*a*_ was non-significant (Δχ^2^_[__2__]_ = 1.49, *p* = 0.47), while M_2__*a*_ improved significantly on M_2__*b*_ (Δχ^2^_[__4__]_ = 14.35, *p* = 0.01). Thus, M_2__*a*_ was our final model to test our hypotheses, with the cross-lagged paths from forgiveness to work outcomes constrained to be equal over time and with their corresponding auto-regressive effects varying over time. As mentioned above, considering the complexity of the cross-lagged model and our relatively small sample size, we treated constructs as observable variables instead of latent variables.

**TABLE 3:** Fit indices for competing models.

	**Model**	**χ^2^**	***df***	**RMSEA**	**CFI**	**TLI**	**SRMR**
M_1_	Stability model	93.52	27	0.13	0.95	0.94	0.08
M_2_	M1 + Forg → WO (CL)	83.26	24	0.13	0.96	0.94	0.05
M_3_	M1 + WO → Forg (reversed CL)	87.08	24	0.14	0.96	0.93	0.07
M_4_	reciprocal model	76.82	21	0.14	0.96	0.93	0.04
M_2__*a*_	M2 + constrain CL to be equal over time	84.75	26	0.13	0.96	0.94	0.05
M_2__*b*_	M2a + constrain AR to be equal over time	99.10	30	0.13	0.95	0.94	0.11

*Forg = Forgiveness; WO = Work outcomes; CL = Cross-lagged effect; AR = Auto-regressive effect.*

**TABLE 4:** Chi-square difference tests of competing models.

**Model**		**Δχ^2^**	**Δdf**	***p***
**Comparison with M1**			
	M_1_ vs. M_2_	10.26	3	0.02
	M_1_ vs. M_3_	6.44	3	0.09
	M_1_ vs. M_4_	16.7	6	0.01
**Equal time lag effects**			
	M_2_ vs. M_2__*a*_	1.49	2	0.48
	M_2__*a*_ vs. M_2__*b*_	14.35	4	0.01

**FIGURE 2:**
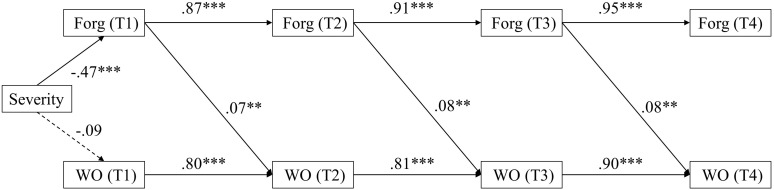
Standardized estimates for significant paths in the forgiveness to work outcomes model. ^∗^*p* < 0.05, ^∗∗^*p* < 0.01, ^∗∗∗^*p* < 0.001 (two-tailed); Forg = Forgiveness; WO = Work outcomes; T1–T4 refer to Time 1–Time 4, respectively (*N* = 130–139).

The results of this final model are displayed in Figure 2. These results revealed that forgiveness predicted an increase in work outcomes from Time 1 to Time 2 (β = 0.07, *SE* = 0.03, *p* < 0.01), from Time 2 to Time 3 (β = 0.08, *SE* = 0.03, *p* < 0.01) and from Time 3 to Time 4 (β = 0.08, *SE* = 0.03, *p* < 0.01). A similar pattern was found when excluding perceived severity as a control variable (see Appendix B). These results provided strong support for our hypothesis that forgiveness facilitates work outcomes.

Moreover, given that we had no theoretical guidelines specifying the length of time for time-lagged effects to be present, we explored the possibility of different time lag intervals and the presence and absence of wave-skipping paths (Meier and Spector, 2013). In particular, we tested whether the hypothesized forgiveness-work outcomes associations still hold when there were 2 weeks or 3 weeks, rather than 1 week, in the time lag. Using the same procedure as outlined above, we included equal cross-lagged effects from forgiveness at Time *i* to forgiveness at Time *i* + 2 (*i* = 1, 2). The results provided adequate model fit (χ^2^ = 88.39, *df* = 26; CFI = 0.96; TLI = 0.94; RMSEA = 0.13; SMRM = 0.06). We found similar findings for a 2-week interval compared to a 1-week interval model (M_2__*a*_). The results revealed that forgiveness predicted an increase in work outcomes from Time 1 to Time 3 (β = 0.07, *SE* = 0.03, *p* < 0.05), and from Time 2 to Time 4 (β = 0.07, *SE* = 0.03, *p* < 0.05). Moreover, we estimated a model whereby forgiveness at Time 1 was set to predict work outcomes at Time 4. The model fit results were acceptable (χ^2^ = 91.18, *df* = 26; CFI = 0.96; TLI = 0.94; RMSEA = 0.13; SMRM = 0.08), while the results showed no significant associations between forgiveness (T1) and work outcomes (T4) (β = 0.06, *SE* = 0.04, *p* = 0.13). Overall, these results indicate that the hypothesized associations only hold when the temporal lag is relatively short (i.e., less than 3 weeks), which means that forgiveness predicts an increase in work outcomes in a relatively short time period.

## Discussion

This study sought to establish the direction of a possible causal link between forgiveness and work outcomes using a longitudinal design with four time points in a sample of working employees. Our findings revealed that the association between forgiveness and work outcomes is causal in nature. While controlling for perceived severity of the incident, forgiving a colleague with whom one has a relatively good work relationship predicts better work outcomes at a later stage. We did not find that work outcomes predict later forgiveness.

First, our study adds to research on forgiveness, especially in the work context. Related to previous studies showing that forgiveness is beneficial in close relationships (Karremans et al., 2003; Bono et al., 2008), our findings revealed that forgiveness is also beneficial in the workplace, as it predicts higher job satisfaction, higher work engagement, and lower burnout. As noted before, an explanation for this may be that a lack of forgiveness affects crucial work relationships. Because the lack of positive, supportive relationships at work has been related to numerous negative outcomes (Day and Leiter, 2014), forgiveness is likely to be associated with increased work outcomes, precisely because forgiveness helps employees to maintain stable work relationships (McCullough, 2000). Furthermore, employees may for various reasons find it difficult to forgive their coworker, despite the good relationship they have. The combination of a lack of forgiveness and the stable and good work relationship may contribute to psychological tension and stress, which may be a second reason for why forgiveness in work relationships reduces work outcomes (for a similar reasoning, see e.g., Karremans et al., 2003; Van der Wal et al., 2016). Finally, it could also be that group-level factors help explain the association between forgiveness and work outcomes. For example, an individual’s (or leader’s) forgiveness may serve as an example for employees on how to deal with conflicts. In this way, interpersonal forgiveness may ultimately create a more forgiving organizational climate (Fehr and Gelfand, 2012), which in turn benefits outcomes in the workplace (Cox, 2011). These findings may inform our understanding of potential mechanisms (even complex feedback loops) of forgiveness and work outcomes in future research.

Second, our study provided compelling evidence that forgiveness resulted in better work outcomes across time, but we did not find evidence for the reverse effect. That is, individual work outcomes did not predict levels of forgiveness to an offending coworker. It could be that such a reversed pattern does not exist. It is also possible that third variables, such as feelings and thoughts, more indirectly help explain the path from work outcomes to forgiveness. For example, people with higher levels of job satisfaction may value their colleagues more, which in turn helps them to forgive (Law, 2013). Moreover, feeling emotionally exhausted at work may increase employees’ negative affect (Little et al., 2007), which in turn makes it more difficult for people to forgive offending others (Fehr et al., 2010). For now, we only found evidence that forgiveness predicts better work outcomes, suggesting that even if the possible reverse indirect paths linking outcomes to forgiveness discussed above are viable, they are considerably weaker than the effects of forgiveness on outcomes.

Third, our findings are based on forgiveness between coworkers in relatively good work relationships. Although we had good reasons for focusing on the role of forgiveness in response to conflicts in particularly good work relationships (Cao et al., in press), the question remains whether forgiveness might also be beneficial in lower-quality work relationships. This is important because, especially in a work context, employees cannot always choose themselves who they work and interact with, including their supervisors, coworkers and subordinates (Day and Leiter, 2014). It is, for example, possible that in low-quality relationships interpersonal offenses are considered the rule rather than the exception (as in high-quality relationships), perhaps (in the work context) leading to higher levels of turnover/withdrawal rather than stress and lower well-being. Given that forgiveness has exclusively been shown to be beneficial in relatively close and stable relationships (Karremans et al., 2003; Bono et al., 2008; Van der Wal et al., 2016), it remains unclear whether our findings can be generalized to offenses that take place in lower-quality work relationships.

Finally, the question remains whether it is *always* good to forgive an offending colleague, even in work relationships of relatively high quality. Although research on forgiveness generally highlights the positive consequences of forgiveness (McCullough et al., 2001; Karremans et al., 2003, 2005), it is important to note that forgiveness might in some circumstances have detrimental outcomes (Luchies et al., 2010; McNulty, 2011; Adams et al., 2015). In particular, in case of repeated offenses and without any signals that one will be safe and valued in the future (such as an apology), forgiving a coworker one has a good work relationship with may go at the cost of an individual’s self-respect and self-concept clarity (Luchies et al., 2010).

### Strengths, Limitations and Future Research

To our knowledge, the present research is among the first to examine the causal associations between forgiveness and general work outcomes. This research used a longitudinal design with four time points among employees working in a variety of different organizations in China. The use of recall methodologies asking participants to recall an incident happened in real organizational settings increased ecological validity (Barclay and Saldanha, 2016). At the same time, this research had several limitations that need to be discussed. First, our sample was predominantly Chinese; thus, it is unclear whether our results generalize among individuals with another cultural background. Moreover, due to the dropout resulting from the four-wave longitudinal design, this research draws on a relatively small sample size. In our analyses, we therefore treated the constructs as observable variables, instead of as latent factors (see also Liu et al., 2020). Nevertheless, it is important that future research replicates our findings using a larger sample across different cultures.

A second limitation is the use of the recall method. First, the accuracy of the recall may be doubtful, as this kind of retroactive reporting could easily be colored by selective memory or current mood or recovery stages (Chi et al., 2019). For example, when participants recalled a hurtful incident from the past, they may have recalled events that they perceived as particularly severe, which could have affected the longevity of the effects found in our study (although identical findings were found when including and excluding perceived severity as a control variable – Appendix B). Moreover, in this longitudinal design, participants were required to recall an offense at Time 1 and reread it at the following study waves. Although unintended, this might have led to increased negative thoughts and feelings, which may have decreased levels of forgiveness (Fehr et al., 2010). We suggest more prospective research to replicate our findings.

A final limitation pertains to the self-report data. Although a longitudinal panel design and randomized presentation of questions may help reduce common method bias (CMB) to some extent (Podsakoff et al., 2003), there is still a possible influence of CMB on the results given that all dependent and independent variables were rated by the same source. Future research should include other measures of forgiveness and work outcomes, such as measuring forgiveness implicitly (IATs) (Goldring and Strelan, 2017), or behaviorally (Dorn et al., 2014). Also, objective data on work outcomes may be used, such as number of days absent, output maintained in organizational records (Koopmans et al., 2011) or subjective judgments from supervisors and peers (Taris, 2006).

### Practical Implications

This research makes a meaningful contribution to the literature because it holds important insights for managers who want to prevent employee burnout and improve employee job satisfaction and work engagement. Our findings indicate that forgiveness might be an essential antecedent of employee well-being, in that forgiveness has a small, yet significant and systematic effect on later work outcomes, controlling for earlier outcomes. On the one hand this underlines the need for managers (and, perhaps, employees themselves) to make sure that conflicts and incidents in the workplace are resolved quickly and effectively, as the adverse consequences of hurtful events tend to linger on for longer periods of time – the negative feelings associated with such events do not seem to go away quickly nor do their effects peter out at short notice, not even in the higher-quality relationships examined in the present study. On the other hand, it is noteworthy for managers that in order to achieve the benefits of forgiveness on employee well-being, the *benefits* of forgiveness are also especially visible in higher-quality work relationships. This indicates that in minimizing the adverse consequences of possibly hurtful events at work, managers might first try to promote connectedness and social relationships between employees as much as possible. This provides a fertile ground to ultimately reap the benefits of forgiveness at work (Struthers et al., 2005).

## Conclusion

Whereas the topic of forgiveness has received much attention in research in social and clinical psychology, only recently scholars started to explore the role of forgiveness in an organizational context. Our research adds to the small and so far exclusively cross-sectional literature on forgiveness at work by showing that forgiveness causally predicts better work outcomes. These findings provide a starting point to further address and promote the topic of forgiveness at work.

## Data Availability Statement

The original contributions presented in the study are included in the article/supplementary material, further inquiries can be directed to the corresponding author/s.

## Ethics Statement

The studies involving human participants were reviewed and approved by the Ethics Committee of the Faculty of Social and Behavioral Sciences of Utrecht University (FETC20-149). The participants provided their written informed consent to participate in this study.

## Author Contributions

WC collected the data and analyzed the results. RW and WC wrote the drafts of the manuscript. TT read the drafts of the manuscript and provided feedback. All authors designed the study, contributed to the article, and approved the submitted version.

## Conflict of Interest

The authors declare that the research was conducted in the absence of any commercial or financial relationships that could be construed as a potential conflict of interest.

## Publisher’s Note

All claims expressed in this article are solely those of the authors and do not necessarily represent those of their affiliated organizations, or those of the publisher, the editors and the reviewers. Any product that may be evaluated in this article, or claim that may be made by its manufacturer, is not guaranteed or endorsed by the publisher.

## Footnotes

1 To minimize potential effects of COVID-19 on our study, we collected data in China from June to July 2020. At that time COVID-19 was under control and working life went back to normal in China (Burki, 2020).

## Appendix A

**TABLE TA1:** Differences between matched samples and unmatched samples.

**Variable**	**Matched samples**			**Unmatched samples**			***t***	***p***
	***M***	***SD***	***N***	***M***	***SD***	***N***		
Forgiveness	4.59	1.23	139	4.64	1.36	358	−0.38	0.71
Job Satisfaction	5.56	0.98	139	5.60	1.16	358	−0.36	0.72
Work Engagement	5.22	1.04	139	5.35	1.19	358	−1.13	0.26
Burnout	2.91	1.30	139	3.12	1.43	358	−1.51	0.13
								

## Appendix B

**TABLE TA2:** Standardized path coefficients of M_2__*a*_ excluding control variable.

		**β**	***SE***	***p***
**Temporal stability effects**			
	Forg (T1) → Forg (T2)	0.87	0.02	0.00
	Forg (T2) → Forg (T3)	0.91	0.02	0.00
	Forg (T3) → Forg (T4)	0.95	0.01	0.00
	WO (T1) → WO (T2)	0.80	0.03	0.00
	WO (T2) → WO (T3)	0.81	0.03	0.00
	WO (T3) → WO (T4)	0.90	0.02	0.00
**Cross-lagged effects**			
	Forg (T1) → WO (T2)	0.07	0.03	0.00
	Forg (T2) → WO (T3)	0.08	0.03	0.00
	Forg (T3) → WO (T4)	0.08	0.03	0.00

*Notes. Forg = Forgiveness; WO = Work outcomes; T1-T4 refer to Time 1-Time 4, respectively.*
